# Calibration in process monitoring by using unsegmented continuous-flow systems

**DOI:** 10.1155/S1463924689000507

**Published:** 1989

**Authors:** M. D. Luque de Castro, M. Valcárcel

**Affiliations:** Department of Analtical Chemistry Faculty of Sciences University of Córdoba Córdoba 14004 Spain

## Abstract

An overview is presented of the different aspects of the role of analytical chemistry in process monitoring and control. On-line monitoring is currently the most attractive option in this area, especially with unsegmented-flow techniques. In addition to allowing automation of these systems, the variety of ways in which calibration and recalibration can be performed allows their adaptation to any situation by using extremely simple, home-made manifolds. The most relevant designs are presented and critically discussed in this paper.


Journal of Automatic Chemistry Vol. 11, No. 6 (November–December 1989), pp. 260–265

### Calibration in process monitoring by using unsegmented continuous-flow systems

**M. D. Luque de Castro and M. Valcárcel**

*Department of Analytical Chemistry, Faculty of Sciences, University of Córdoba, 14004 Córdoba, Spain*

*An overview is presented of the different aspects of the role of analytical chemistry in process monitoring and control. On-line monitoring is currently the most attractive option in this area, especially with unsegmented-flow techniques. In addition to allowing automation of these systems, the variety of ways in which calibration and recalibration can be performed allows their adaptation to any situation by using extremely simple, home-made manifolds. The most relevant designs are presented and critically discussed in this paper.*

### Introduction

Traditionally, the analytical chemist's responsibility has been limited to the laboratory. Recently their rôle has stopped starting at the laboratory door and finishing at the printer or plotter. Analytical chemists are currently involved in such tasks outside the laboratory as sampling, and establishing a rapport with other scientists/technicians to promote, perform and evaluate basic and applied developments. One of these tasks, process control, is a target at which the endeavours of analytical chemists should be aimed in the forthcoming years.

There are several reasons for the growing importance of process analytical chemistry. Firstly there is economics, no doubt the most significant; and then there are technological reasons. Increasing demands are imposed on the quality of raw materials and intermediate and end products in all kinds of processes, this is because quality determines their potential technical use to a very great extent. Any process optimization requires reliable, fast and precise control systems.

Until recently, analytical measurements in production processes were the responsibility of process or systems engineers. The signal provided by the measuring instrument was often simply used for the adjustment of operational parameters without interpreting it for checking or calibration purposes. The present growing demand for quality makes analytical chemists essential to the production process. As a result, process analysis has become a substantial part of analytical chemistry. For example, in the USA the CPAC (Center for Process Analytical Chemistry) has recently been set up supported by the National Science Foundation and aimed at the development of analytical methods for direct integration into the production process and coupling with chemometric techniques [1]. The issues involved in the basic research on process analysis and control carried out at the CPAC are:

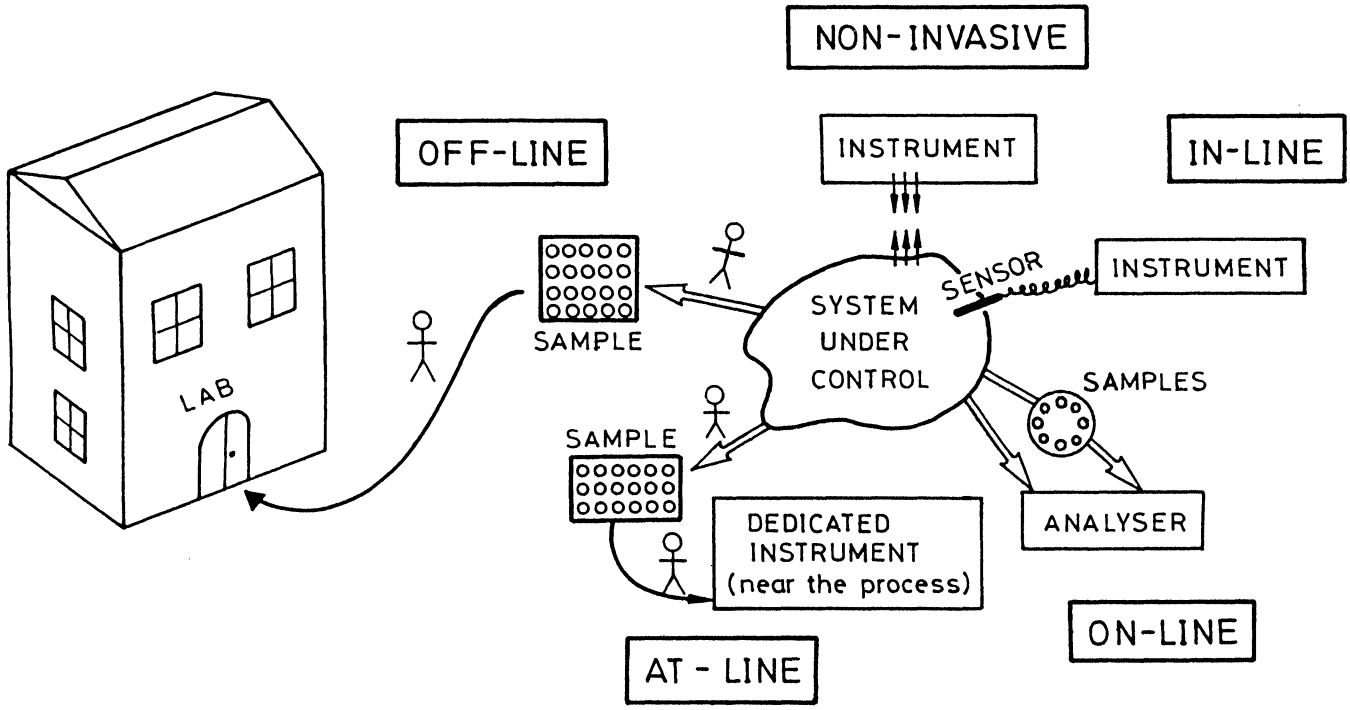

*Figure 1. Different alternatives to process monitoring.*

0142–0453/89 $3.00 © 1989 Taylor & Francis Ltd.

(1) Sampling (contamination, process disruption, frequency, duration, carry-over etc.).
(2) Design of suitable instrumentation (especially chemical sensors).
(3) Development of multivariate data-processing methods to enhance such analytical factors as the calibration range, resolution, precision, sensitivity and selectivity.
(4) Automation and control (automated error detection and correction, optimum control algorithms, artificial intelligence etc.).

In recent papers Callis *et al.* [2] and van der Linden [3] presented the state of the art in process analytical chemistry. There are two general approaches to it which depend on the location of the measuring instrument and the degree of human participation:

(*a*) Traditional off-line monitoring, in which the conventional instruments are all in the laboratory, and the human operators are highly involved in the process (low automation).
(*b*) Use of process analysers (these are dedicated instruments located near the monitored system). Depending on the way in which these analysers are integrated into the process, measurement procedures can be classified into at-line, on-line, in-line and non-invasive.

Figure 1 shows a scheme of all the variants of process analytical chemistry.

In off-line monitoring, sampling and sample transport are performed with a high degree of human participation.

In at-line analysis, sampling is performed manually and the samples are rapidly transported to a dedicated instrument which is installed in the vicinity of the monitored system (for example, natural system process unit, process line etc.).

In on-line monitoring human participation is strongly reduced. Sampling and sampling conditioning are completely automated and are an integral part of the analysed system. Sampling is primarily done by the continuous changing of an aliquot of the liquid or gas from the process and it is the chief source of problems in on-line analysers.

In-line analysers require no conventional sampling. A selective device is placed in direct contact with the system under control. This is a very promising approach, despite such major drawbacks as: the small number of suitable sensors currently available: the impossibility of conditioning samples; the need to remove the device from the process for calibration and the fact that the fouling of the sensor can cause base-line drifts, masking of the response, or even complete disappearance of the signal.

In non-invasive process anlysers there is no physical (mechanical) contact between the sample and probe (or instrument). Thus, sampling problems are greatly reduced. These are the most desirable analysers but they are still in an early stage of development and application.

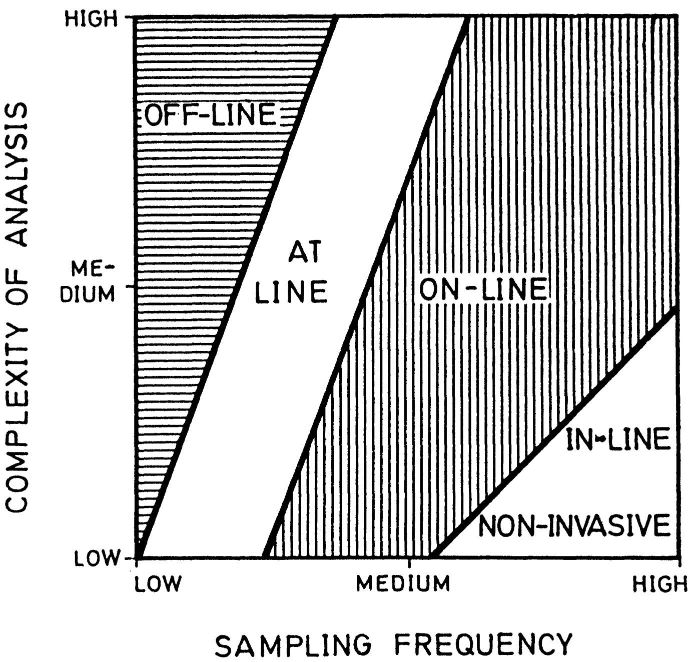

*Figure 2. Use of various types of approaches in relation to sampling frequency and analysis complexity.*

The choice between the different approaches to process analysis will depend on the complexity of the analysis (number of monitored analytes, use of derivatization reactions, need to dilute, sample clean-up, and the sampling frequency dictated by the features of the monitored process). Figure 2 shows a graphical approach to the different fields of application in process analytical chemistry.

When the complexity of analyses is high and the sampling frequency is low, off-line monitoring is the best choice. However, if analyses are very simple and a high sampling frequency is required, the newer alternatives (in-line and non-invasive) are the best options. The at-line alternative has a narrow scope of application, compared with the on-line approach, which is currently the most attractive for continuous or intermittent monitoring and control purposes, provided that sampling is representative and reproducible.

Calibration is a fundamental step in process monitoring and its complexity depends on the particular variant selected.

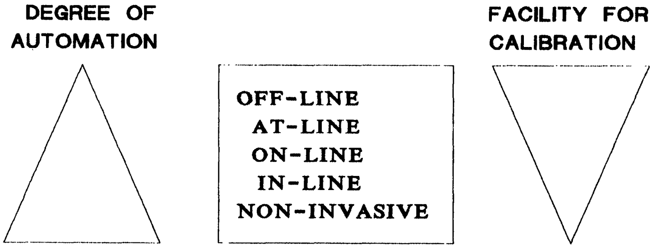

*Figure 3. Automation capabilities and ease of calibration of the analysers used for process monitoring.*

Continuous unsegmented-flow analytical methodologies adapted to the monitoring of an evolving system make the best alternative to on-line analysers [4, 5]. The different approaches to on-line calibration can be classified according to whether or not they involve any disruption of the monitoring process.

Calibration with disruption in on-line monitoring can be performed by using unsegmented-flow configurations in completely continuous flow analysis (CCFA) [6], or by normal, or reversed, flow injection analysis [7]. Calibration without disruption monitoring is a more appropriate alternative when a high sampling frequency is required, and can be implemented on hybrid CCFA-FIA configurations [8] or on simple FIA manifolds with suitable injection systems [9–11].

### Calibration with monitoring disruption

Figure 4 shows the three types of configurations typically used for this purpose.

The completely continuous configuration (figure 4[*a*]) requires a selecting valve which allows standards to be introduced into the system at appropriate time intervals, which in turn are a function of the features of the detector (risk of poisoning, or fouling, and adsorption phenomena with electroanalytical detection and base-line instability with others). The faster calibration demanded by rapidly evolving sysems can be accomplished by using four or five standards each working day, and another one or two standards for intermediate recalibration.

Normal and reversed flow injection configurations feature a similar degree of complexity. The use of one or the other will be dictated by the features of the system to be controlled and those of the derivatizing reaction used for monitoring. Normal FIA systems (n-FIA) are more advantageous for scarce or expensive samples and inexpensive reagents, while reversed FIA (r-FIA) manifolds are to be preferred for abundant, affordable samples and expensive reagents. One possible solution when both samples and reagents are scarce or expensive is the use of a dual valve for the simultaneous injection of the sample (or standard) and reagent [12]. In any case, recalibration can be performed with a single standard, with better precision than that provided by completely continuous configurations, because the base-line is checked between successively injected samples.

### Calibration without monitoring disruption

When the changes in the process line are both fast, disrupting, monitoring for calibration may pose serious problems. These can be overcome by using a CCFA-FIA configuration, or, even better, by using injection systems

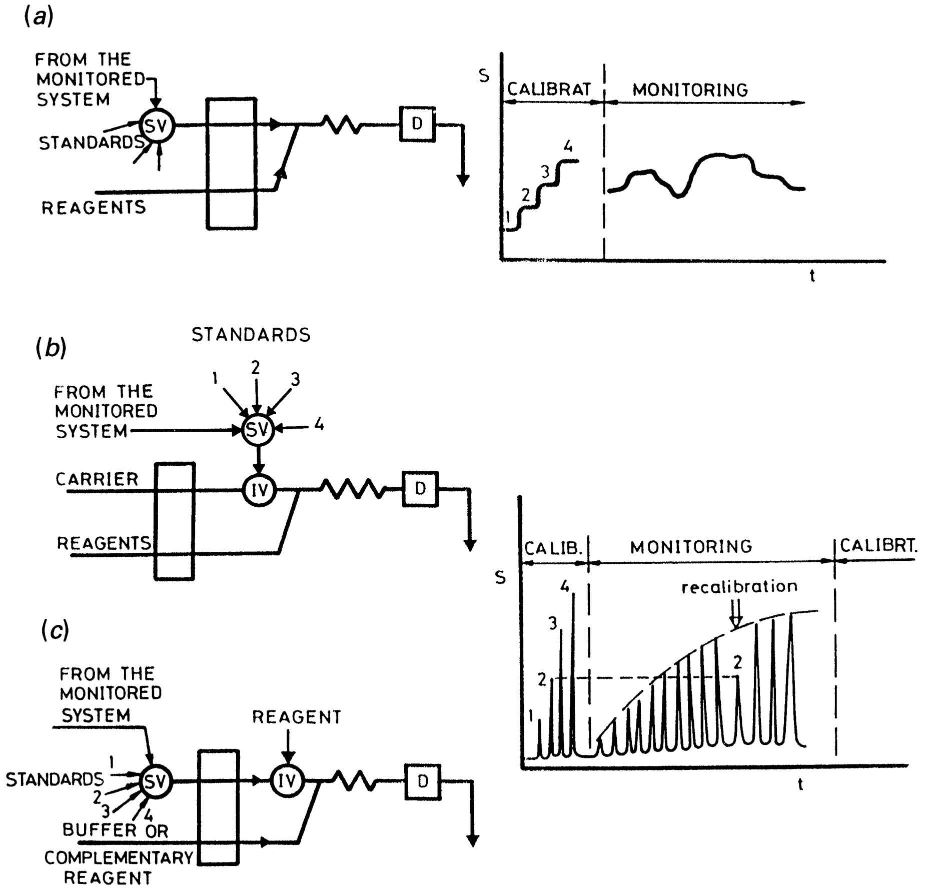

*Figure 4. Straightforward unsegmented-flow configurations for on-line process monitoring and calibration. (a) Completely continuous. (b) Normal FIA; Reversed FIA. SV and IV denote selecting and injection valve, respectively, D detector, S analytical signal and t time.*

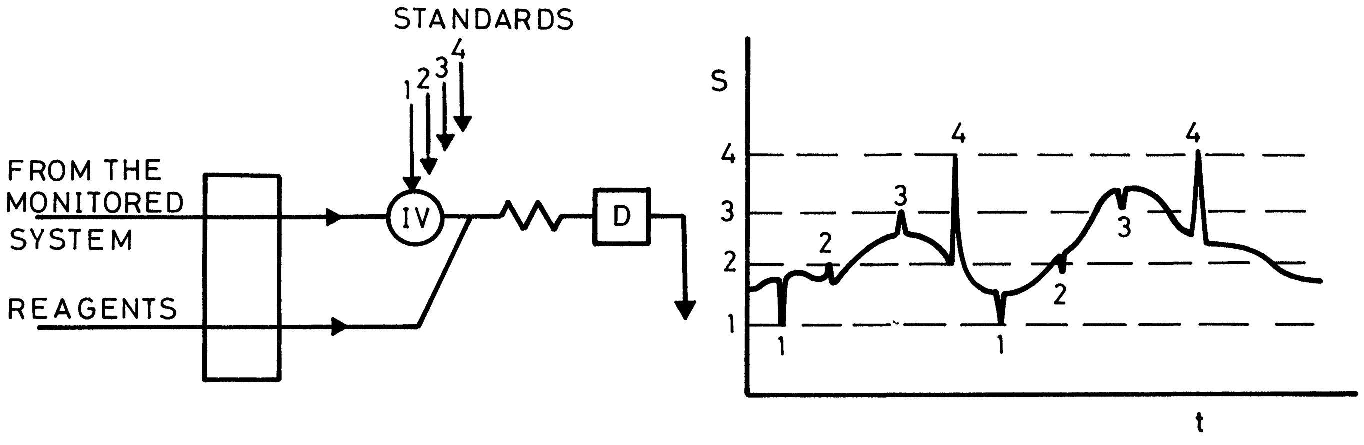

*Figure 5. Configuration with continuous monitoring of the process and sporadic calibration.*

which allow calibration to be made simultaneously with the measurement of the analyte. An eight-port, a six-port injection valve or an internally coupled valve system can be used for this purpose.

The hybrid configuration (figure 5) involves the continuous introduction of the sample into the system and the use of a conventional injection valve to insert the standards at preset times. This operational mode, proposed by Frenzel and called 'modified reverse FIA' [8], is actually more of a hybrid between the completely continuous technique (continuous mixing of the sample and reagent to provide continuous recording, in contrast with the transient signals obtained with r-FIA configurations). The use of this configuration introduces two major sources of error; (1) the absence of base-line checking, which can result in spurious changes in the concentration of the monitored analyte [4, 5, 8]; and (2) errors in the standard signal arising from its insertion into a stream containing a variable concentration in the process line [13].

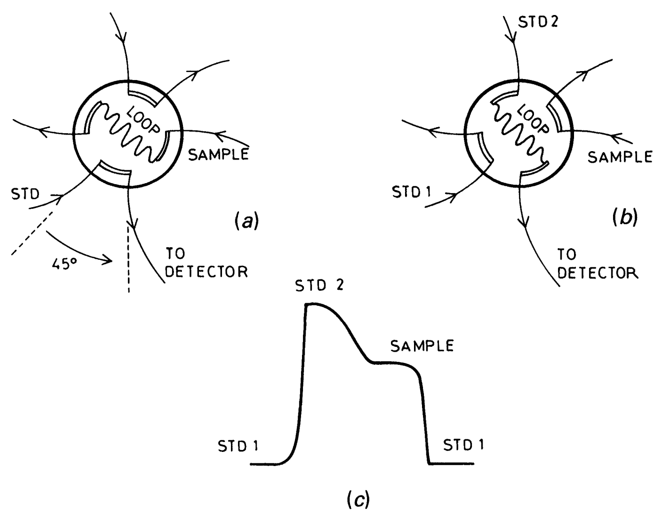

*Figure 6. Eight-port injection valve for calibration without monitoring disruption. (a) Load position. (b) Inject position. (c) Recording. SDT1 and STD2 are standards.*

The following procedures for calibration involving special valves, or the coupling of conventional valves, are called 'sandwich procedures' because the sample plug is placed between two plugs of standard at the same or at a different concentration.

Alonso *et al.* [9] used a home-made eight-port injection valve, which can be switched between the load and inject positions shown in figure 6, thus intercalating the sample plug between two different standards, one of which acts as carrier (STD1) and the other (STD2) being simultaneously injected with the sample. In this way, the base-line is established by STD1 and the recording consists of two plateaux corresponding to the sample and STD2, respectively (figure 6[*c*]). Consumption of standards is very high.

The six-port valve (figure 7), proposed by Ruzicka and Hansen [10], allows the sample to be inserted between two plugs of the same standard, thus providing the profile shown in figure 7(*c*); the recordings obtained will be of d1 type if the analyte concentration in the standard is higher than that of the sample, or of d2 type, if the opposite holds.

An internally coupled valve system [14] also allows for continuous automatic calibration by using the secondary valve for sample insertion and the two sub-loops of the main valve for the standard. Figure 8(*a*) show the manifold use, and figure 8(*b*) shows the operation of the injection system, which after injection provides a profile along the reactor similar to that obtained by the procedure described above (see figure 7[*c*]). Three signals similar to those shown in figure 7(*d*) are provided by each injection; the central one, corresponding to the sample, is flanked by those corresponding to the standard.

Approaches involving special injection systems have two serious drawbacks:

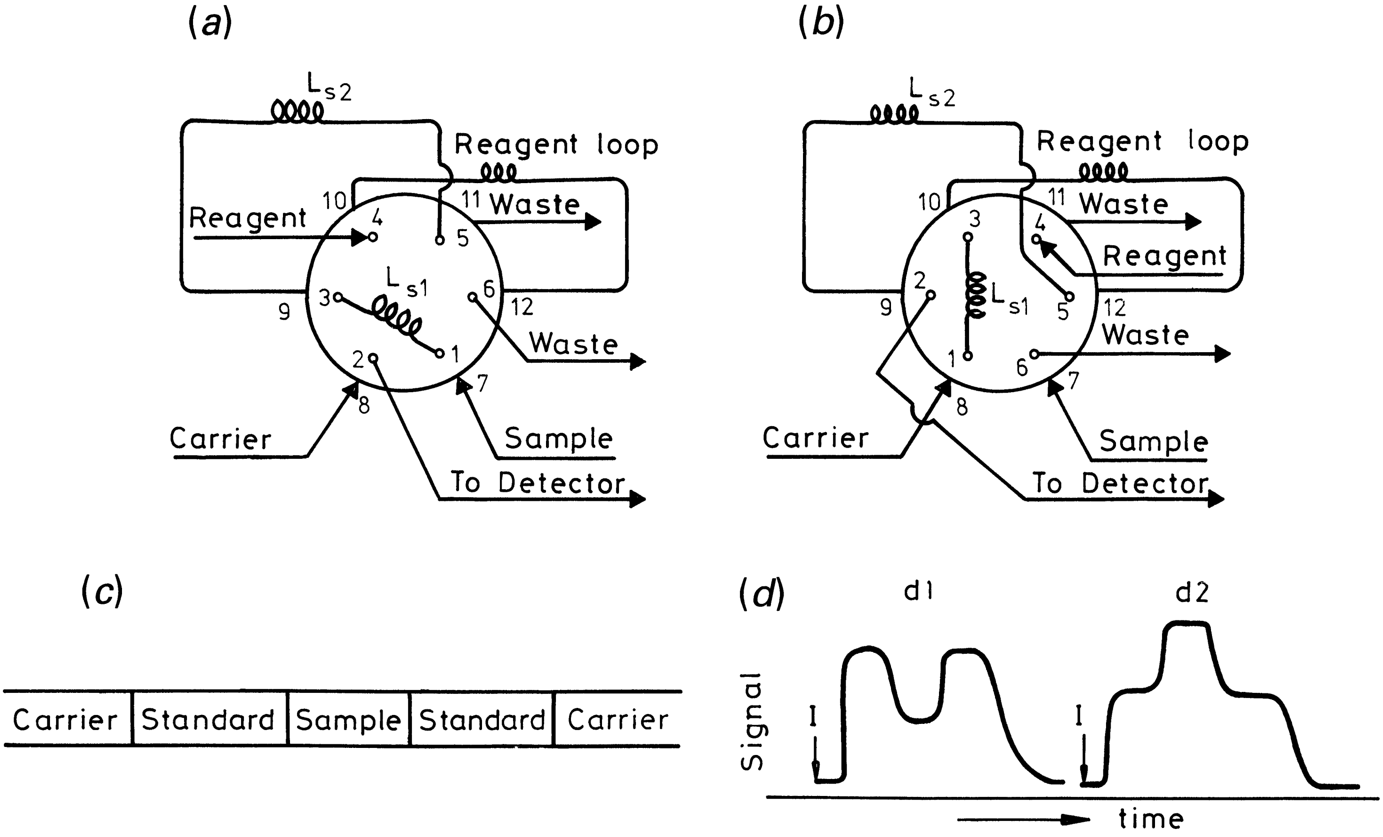

*Figure 7. Calibration without monitoring disruption with a six-port injection valve. (a) Load position. (b) Inject position. (c) Profile of the injected solutions along the transport system. (d) Recordings d1 and d2.*

(1) High sample and standard consumption per injection (abnormally in FIA and of the order of 0·5 to 1·0 ml), because a non-mixing zone between sample and standard is required in the head and tail, respectively, of the overall plug injected, in order to obtain well-defined plateaux from each, thus avoiding the above mentioned problem of variable dispersion in the hybrid configuration.

(2) Standard consumption is very high, taking into account the continuous calibration and the fact that this type of calibration is used whenever a high sampling frequency is required. This problem can be overcome by using a selecting valve prior to the valve injecting the standard, allowing the standard stream to be replaced with a carrier stream when no calibration is required. This selecting valve also

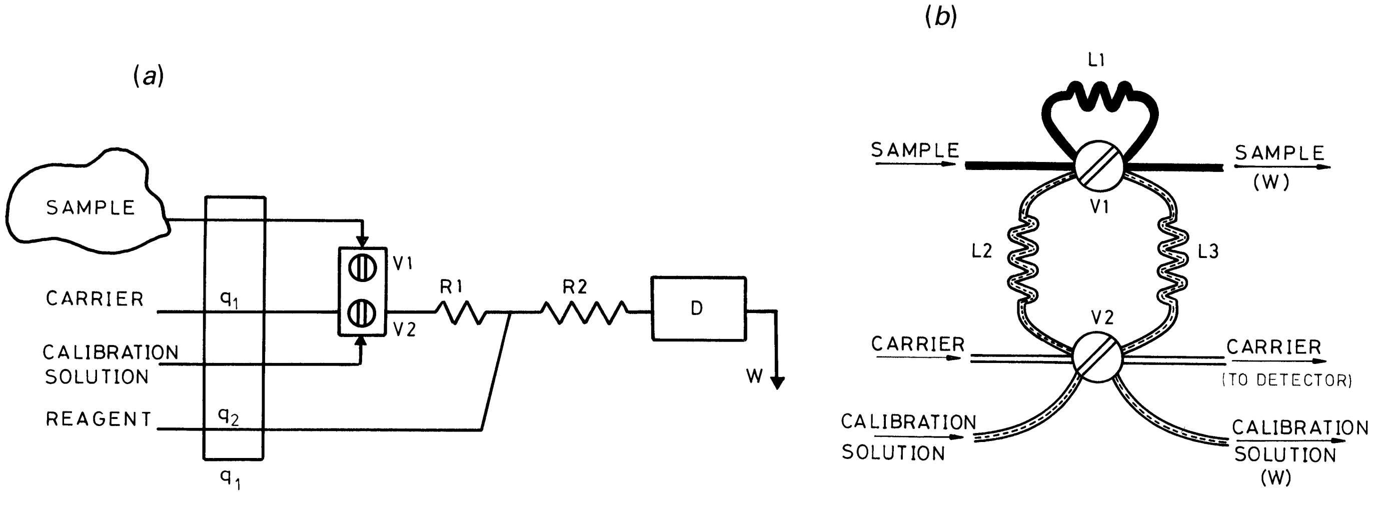

*Figure 8. Calibration without monitoring disruption with internally coupled valves. (a) FIA configuration. (b) Valve coupling. q flow-rate, V1 and V2 injection valves, R1 and R2 reactors, D detector, w waste, L1, L2, L3 loops.*

permits the concentration of standard to be automatically changed by switching between different streams.

### Final remarks

Unsegmented flow techniques offer interesting possibilities in the field of on-line process monitoring; they use straightforward, inexpensive instrumentation, feature real-time responses and facilitate calibration and recalibration.

The possibilities described in this paper for automatic on-line calibration and recalibration can and should be expanded: the potential of unsegmented flow techniques in this area only depends on the imagination of users to design new systems for introduction of samples or new uses for available systems.

### References

1. Callis, J. B., Illman, D. L. and Kowalski, B. R., Analytical Chemistry, **59** (1987), 624A.
2. van der Linden, W. E., *Analytica Chimica Acta,* **216** (1989), 307.
3. Illman, D. L., *Trends in Analytical Chemistry,* **5** (1986), 164.
4. van der Linden, W. E., *Analytica Chimica Acta,* **179** (1986), 91.
5. Luque de castro, M. D., *Talanta,* **36** (1989), 591.
6. Goto, M., *Trends in Analytical Chemistry,* **2** (1983), 92.
7. Valcárcel, M., and Luque de Castro, M. D., *Flow Injection Analysis: Principles and Applications* (Ellis Horwood, Chichester, 1987).
8. Frenzel, W., and Fresenius Z., *Analytical Chemsitry,* **329** (1988), 668.
9. Alonso, J., Bartrolí, J., del Valle, M., Escalada, M., and Barber, R., *Analytica Chimica Acta,* **199** (1987), 191.
10. Ruzicka, J., and Hansen, E. H., *Flow Injection Analysis* (Wiley, New York, 1988).
11. Rios, A., Luque de Casrto, M. D., and Valcárcel, M., *Talanta,* **36**(5) (1989), 612.
12. Zagatto, E. A. G., Krug, F. J., Bergamin, H. F$^{o}$., Jorgensen, S. S. and Reis, B. F., *Analytica Chimica Acta,* **104** (1979), 279.
13. Chen, Danhua, Luque de castro, M. D. and Valcárcel, M., *Analytica Chimica Acta* (in press).
14. Ríos, A., Luque de Castro, M. D. and Valcárcel, M., *Analytical Chemistry,* **58** (1986), 663.

